# Triple Co-Administration of Ivermectin, Albendazole and Praziquantel in Zanzibar: A Safety Study

**DOI:** 10.1371/journal.pntd.0000171

**Published:** 2008-01-23

**Authors:** Khalfan A. Mohammed, Hamad J. Haji, Albis-Francesco Gabrielli, Likezo Mubila, Gautam Biswas, Lester Chitsulo, Mark H. Bradley, Dirk Engels, Lorenzo Savioli, David H. Molyneux

**Affiliations:** 1 Programme for Lymphatic Filariasis, Schistosomiasis and Soil-Transmitted Helminthiasis, Ministry of Health and Social Welfare, Zanzibar, United Republic of Tanzania; 2 Lymphatic Filariasis Support Centre, Liverpool School of Tropical Medicine, Liverpool, United Kingdom; 3 Public Health Laboratory Ivo de Carneri, Chake Chake, Pemba Island, Zanzibar, United Republic of Tanzania; 4 Department of Neglected Tropical Diseases (NTD), World Health Organization, Geneva, Switzerland; 5 Other Tropical Diseases (OTD), World Health Organization Regional Office for Africa (WHO/AFRO), Belvedere, Harare, Zimbabwe; 6 Global Community Partnerships, GlaxoSmithKline, Brentford, United Kingdom; Swiss Tropical Institute, Switzerland

## Abstract

**Background:**

Public health interventions based on distribution of anthelminthic drugs against lymphatic filariasis (LF), onchocerciasis, soil-transmitted helminthiasis (STH) and schistosomiasis have been implemented separately to date. A better use of available resources might be facilitated by a more coordinated approach to control such infections, including the possibility of co-administering the three recommended anthelminthic drugs through a single, large-scale intervention.

**Methodology/Principal Findings:**

Ivermectin, albendazole and praziquantel were co-administered to 5,055 children and adults living in areas endemic for LF, STH and schistosomiasis in Zanzibar, United Republic of Tanzania, during a pilot intervention aimed at elucidating and quantifying possible side-effects. Subsequently, these drugs were co-administered to about 700,000 individuals during a countrywide intervention targeting a large part of the total population of Zanzibar. Passive and active surveillance measures carried out during both interventions showed that side-effects attributable to the three drugs given at the same time were mild and self-limiting events.

**Conclusions/Significance:**

Our data suggest that co-administration of ivermectin, albendazole and praziquantel is safe in areas where lymphatic filariasis, soil-transmitted helminthiasis and schistosomiasis are co-endemic and where several rounds of treatment with one or two drugs have been implemented in the past. Passive surveillance measures, however, should be continued and detection, management and reporting of possible side-effects should be considered a key component of any health intervention administering drugs.

## Author Summary

This paper describes how the use of three drugs which are used separately in mass drug distribution programmes when given together appear safe for use in large populations which have been previously treated with the same drugs separately (Mectizan [ivermectin], albendazole and praziquantel). The target diseases—lymphatic filariasis, soil-transmitted worms and schistosomiasis—were prevalent in Zanzibar up to 2000 but have been largely controlled by mass drug administration. The Ministry of Health and Social Welfare, with the support of WHO, initiated a small scale trial in a population of triple therapy in over 5,000 people initially in two sites, and having found there were no severe adverse events associated with the combined treatment then upscaled to treat the whole of the eligible population of over 700,000. Similarly, there were no severe adverse events. This is the first time the three drugs have been used together at the same time at scale in Africa and provide a basis for expansion of integrated preventive chemotherapy of helminths (worms). The next steps need to be initiated in populations which have heavier worm loads and such interventions need to be subject to close monitoring and ethical review.

## Introduction

Lymphatic filariasis (LF), soil-transmitted helminthiasis (STH) and schistosomiasis are diseases of considerable public health importance in tropical and sub-tropical countries. Globally, 1.2 billion people live in areas endemic for LF [1] and nearly one-fourth of them may already have infection [2]. LF is a leading cause of long-term disability [3]. Schistosomiasis occurs in over 70 countries in the tropics and sub-tropics; 779 million are estimated to be at risk of infection and 207 million to be infected [4]–[6]. 2 billion are estimated to be infected with STH, namely the roundworm (*Ascaris lumbricoides*), the whipworm (*Trichuris trichiura*), and the hookworms (*Ancylostoma duodenale* and *Necator americanus*), worldwide and several million suffer from the chronic debilitating morbidity [7]–[10].

In most endemic countries, these infections often occur in the same individual [11]. This is especially true in poorest sectors of the population. Helminth infections have a substantial impact on the physical and intellectual development and on the overall health status of infected populations, as well as on the quantity and quality of their productive work [12]–[16]. Interventions to tackle helminth infections and their associated morbidity have been taken by national programmes in different countries including Zanzibar, in the United Republic of Tanzania; however such activities are implemented in a vertical fashion, with different diseases being addressed separately.

### Control of LF, STH and Schistosomiasis in Zanzibar

Zanzibar comprises 2 main islands, Unguja and Pemba, with a population of around 1.2 million. LF, caused by *Wuchereria bancrofti,* and STH and schistosomiasis are considered major public health problems by the Zanzibar Ministry of Health and Social Welfare (MoHSW). Transmission of *W. bancrofti* occurs in both Unguja and Pemba [17]. Such an epidemiological situation justifies the inclusion of both islands among the areas eligible for mass drug administration (MDA) with ivermectin and albendazole at yearly intervals. Data show that the whole archipelago is also at high risk for STH; with the exception of a limited area on Unguja where the infection is not transmitted, it is also at high risk for urinary schistosomiasis caused by *Schistosoma haematobium* (MoHSW unpublished data, [18]–[21]). Control of schistosomiasis and STH through large-scale preventive chemotherapy interventions distributing praziquantel and albendazole started in 1994. Schools represented the main delivery channel and schoolchildren the main target population, however, occasionally whole communities have been also targeted for treatment. This strategy is still on-going with a period of interruption of two years (2000 and 2001) during which activities did not take place due to problems in securing the drugs. There has been a reduction in prevalence and intensity of both schistosome and STH infections over the years (MoHSW unpublished data), however such indicators suggest that continuation of treatment of schoolchildren is still required. The last school-based drug distribution before the implementation of the activities described in this survey was carried out in May 2006.

In 2001 Zanzibar adopted the WHO recommended strategy against LF, consisting of MDA for elimination as a public health problem, coupled with disability prevention and management. MDA in Zanzibar involves administering a once-yearly dose of a combination of ivermectin (Mectizan, 200 µg/kg) and albendazole (400 mg) to its entire population, with the exception of those who are sick or infirm, of children <90 cm in height, of pregnant women, and of lactating women in the first week after birth. The last round before the implementation of the activities described in this article was conducted in August 2005 (5^th^ round of MDA) [17].

*W. bancrofti* was highly endemic in both Unguja and Pemba before MDAs, with a prevalence of microfilaraemia in all age groups (children and adults) ranging between 5% and 30% [17]. Whilst the evaluation of the impact of MDAs showed an overall decline in both prevalence and intensity of microfilaraemia, mean prevalence of infection in all age groups (adults and children) at one sentinel and in some randomly selected spot-check sites after the 5^th^ round was still 1% and above. The MoHSW therefore decided to implement a further round (6^th^) of MDA. This follows the WHO recommended processes leading to decision to stop MDA only after interruption of transmission (prevalence of microfilaraemia <1% in the general population) [22].

### Triple Drug Co-Administration

The 6^th^ round of MDA for LF offered the opportunity to evaluate the feasibility and safety of triple drug co-administration with ivermectin, albendazole and praziquantel in communities where LF, STH and schistosomiasis are co-endemic.

Ivermectin, albendazole and praziquantel are well suited for large-scale distribution in helminth control or elimination when administered individually or in double combination (ivermectin and albendazole *or* albendazole and praziquantel) at the recommended dosages (ivermectin 200 µg/kg; albendazole 400 mg; praziquantel 40 mg/kg). Combination of ivermectin and albendazole is highly efficacious after a single administration for LF, and treatment rarely results in side-effects outside those commonly associated with a therapeutic effect [23]. Similarly, co-administration of praziquantel and albendazole is an efficacious and safe tool to control morbidity due to schistosomiasis and STH in areas where both infections are endemic [24].

However, side-effects do sometimes occur following administration of anthelminthic drugs, primarily as a result of the individual's immune inflammatory response to dying parasites; the greater the infection load in the patient, the greater are the frequency and severity of such reactions. These can include systemic responses (e..g. headache, myalgia, light-headedness, anorexia, malaise, nausea, vomiting and wheezing, abdominal discomfort, dizziness, drowsiness, rectal bleeding); or, less commonly, localized reactions (including lymphadenitis, funiculitis, epididymitis, lymphangitis and even abscess formation); rarely, in case of administration of praziquantel for schistosomiasis, hypersensitivity reactions, fever, pruritus and eosinophilia may occur. Only seldom (in heavily infected individuals) are these post-treatment reactions severe or do they require more than just symptomatic treatment [25].

Triple co-administration of ivermectin, albendazole and praziquantel has never been carried out in large-scale interventions, however, pharmacokinetic studies performed in healthy (i.e. non-infected) individuals indicate that there are no pharmacological interactions between the three drugs and that triple co-administration does not enhance their toxicity [26].

However, because of the lack of documentation on such triple co-administration in real epidemiological scenarios, and considering that in those settings some of the individuals receiving drugs may carry high burden of multiple parasites, it is recommended that triple co-administration is carried out with caution and with adequate monitoring of potential side-effects [1]. As a first step it is advisable that in a population that has never been subjected to MDA with any of these drugs, the initial 1–2 rounds of treatment with praziquantel should be given separately from ivermectin and/or albendazole treatment; additionally, in a population that has previously been subjected to (separate) MDA with either ivermectin plus praziquantel or ivermectin+albendazole plus praziquantel, the three drugs should be co-administered in conjunction with additional safety monitoring for any unanticipated side-effects during the initial rounds.

Since in Zanzibar separate MDAs had already been conducted in the past, it was decided to implement co-administration of the three drugs with the requisite precautionary measures. Before co-administering the drugs to the entire eligible Zanzibar population of around 1 million, it was therefore considered imperative that such intervention take place in a pilot population and that active and passive surveillance measures be implemented during and after treatment.

Results of the pilot intervention were considered crucial to the initiation of a country-wide intervention. It was agreed that if side effects during the pilot intervention were mild and transitory, co-administration would take place throughout Zanzibar and that active and passive surveillance measures would be also implemented during such a country-wide interventions.

The aim of the present paper is to report on the outcome of passive and active surveillance measures carried out to elucidate and quantify any side-effects experienced after co-administration of ivermectin, albendazole and praziquantel by a sample population of 5,055 and subsequently by about 700,000 individuals living in areas endemic for LF, STH and schistosomiasis in Zanzibar.

## Methods

### The Pilot Intervention Study Area

The pilot intervention was conducted in 2 highly endemic sites, one from each of the two islands forming Zanzibar: Kinyasini on Unguja (resident population of approximately 4,000) and Mtambile on Pemba (resident population of approximately 3,000).

Field assessments conducted in all age-groups before the survey in early November 2006 showed that at Kinyasini prevalence of LF antigenaemiaia (assessed by an ImmunoChromatographic Test ICT) [27]) was 4.0%, of urinary schistosomiasis (assessed by urine filtration) was 63.5% and of STH infections (assessed by Kato-Katz method) [28] was 76.8%; at Mtambile, these figures were 13.0%, 43.0% and 73.0%, respectively (MoHSW unpublished data). The major occupations of the community in both sites are linked to agriculture [29]. Both sites are surrounded by permanent water bodies.

### Criteria for Eligibility

The whole population of both Kinyasini and Mtambile was considered for enrolment in the pilot intervention. Criteria for enrolment coincided with criteria for eligibility for administration of ivermectin and albendazole in LF disease-specific interventions, which are the most restrictive among the criteria used in interventions against one of the three diseases (LF, STH, and schistosomiasis) taken singularly. As such, we considered eligible all consenting residents of both sites, with the exception of those who were sick or infirm, of children <90 cm in height, of pregnant women, and of lactating women in the first week after birth. All consenting participants (written and oral) were interviewed in order to determine their health status before the intervention, and all their information were recorded.

### Drugs and Their Administration

All participants in the study were given their respective dosages of ivermectin (200 µg/kg), albendazole (400 mg) and praziquantel (40 mg/kg) at the same time; treatment was directly observed by drug distributors to ensure that tablets were actually swallowed. The dosage for ivermectin and praziquantel was calculated using a two-sided tablet (height) pole that was the combination of the two poles currently used for separate administration of such drugs [1], [30]–[32]: one side for determination of the number of ivermectin tablets and the other side for the number of praziquantel tablets. The two sides were clearly made distinguishable by the presence of the Kiswahili (local language) text “kichocho” (schistosomiasis) on one side and “matende” (elephantiasis, LF) on the other. One tablet of albendazole (400 mg) was used as the standard dose for everybody irrespective of age or height [1].

Theoretical and practical training sessions were held during the weeks preceding the intervention so as to train drug distributors - who were familiar with single-drug poles - on the use of the two-sided poles and on the shape and strength of each drug administered, so as to avoid any risk of miscalculation of the dose of ivermectin and praziquantel to be administered.

The strategy of drug distribution and the individuals responsible for drug distribution were the same used in those two sites during previous interventions of MDA with ivermectin and albendazole for elimination of LF.

The pilot intervention took place on 18 November at Kinyasini 2006 and on 19 November 2006 at Mtambile.

### Surveillance Measures

Both passive and active surveillance measures were implemented during the pilot intervention. Passive measures were aimed at ensuring rapid medical assistance for any individuals who might experience side-effects after treatment, while active measures were in place to elucidate the nature of and quantify any such event.

Passive measures were established on the treatment day and the day after; two health centres at Kinyasini and two at Mtambile were kept open round the clock and equipped with first-line emergency drugs (non-steroid anti-inflammatory drugs, anti-histaminic drugs, cortisone, intravenous fluids). Individuals who received drugs were invited to report to these first-line centres in the event of any side-effects (“anything abnormal occurring in your body”), health centre personnel were trained on how to fill in the record forms and instructed to refer to second-line hospitals any individual presenting with side effects that they could not manage. In addition, vehicles/motor bikes patrolled the area to facilitate a rapid response should it be needed.

Active surveillance measures were also carried out between the 5^th^ and the 7^th^ day after treatment. All treated individuals were interviewed on occurrence of side-effects by experienced professional health staff and/or researchers who had participated in previous health survey studies done in the country and who were not resident either in Kinyasini or Mtambile. The occurrence of any side-effects following the ingestion of the three drugs was investigated. A side-effect was defined as any abnormal event experienced by a treated individual within period of observation. A structured questionnaire listing the most common symptoms and signs usually reported after administration of anthelminthic drugs was used to record events, but these events were not read to the interviewee who was asked the following question: “How have you been feeling since taking the treatment?” and left free to answer and to grade the possible event as mild, moderate or severe. For standardization of the approach to be used when administering the questionnaires,health staff/ researchers received one day training, on how to ask the questions and fill up the form.

### Ethical Clearance

The protocol of the pilot intervention was reviewed and approved by the Ethical Committee of the Liverpool School of Tropical Medicine, United Kingdom, and by the MoHSW, Zanzibar. All individuals participating in the pilot intervention or their parent/guardian in case of children provided a written informed consent to treatment. All records are available for scrutiny in the MoHSW, Zanzibar. The procedure regarding the administration of questionnaires to designated interviewees was the same for the pilot project and the nationwide intervention. The individuals gave consent prior to being interviewed for adverse events. It is the policy of the MOHSW to obtain consent from involved communities or involved individuals before carrying out activities in the field.

## Results

### Population under Study

**Figure 1:**
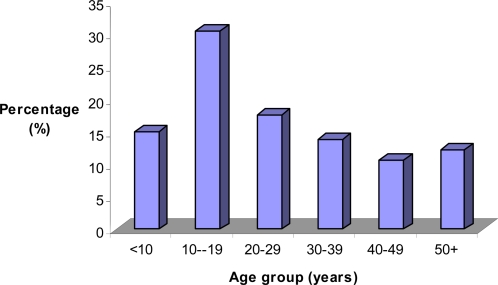
Population participating in the pilot intervention.

A total of 5,055 individuals of both sexes aged 5 years and above, participated in the pilot intervention: 2,509 from Kinyasini and 2,546 from Mtambile. The age-group 10–19 years old was the most represented, in line with current demographic trends in Tanzania and most developing countries [27]. The number of females was higher than that of males (Table 1, Figure 1). This can be mainly explained by the fact that both Kinyasini and Mtambile are rural areas where young males are either farmers or have employment in town. Hence, they usually leave their houses early in the morning and return back late in the evening.

**Table 1:** Population participating in the pilot intervention.

		Treated population	
Age group	Total	Female	Male
<10	763 (15.1%)	455	308
10–19	1548 (30.6%)	885	663
20–29	890 (17.6%)	609	281
30–39	696 (13.8%)	477	219
40–49	538 (10.6%)	327	211
50+	620 (12.3%)	398	222
**Total**	**5055 (100%)**	**3151 (62.3%)**	**1904 (37.7%)**

### Passive Surveillance Measures

Only 1 treated individual reported to a first-line health centre (in Kinyasini) complaining about vomiting that started following treatment; symptoms were, however, mild and successfully managed on spot without requiring referral to a second-line hospital facility.

### Active Surveillance Measures

The results of the interviews carried between day 5 and 7 post triple drug administration are shown in Table 2. Overall, a total of 615 events were reported by 504 individuals, i.e. by approximately 10% (504/5055) of those treated. Occurrence of side-effects was the same (10%) in different sexes and peaked in the age-group 30–39 (Table 2). 87.3% of symptoms occurred within 24 hours of treatment, while a few were also reported to have occurred on the second (11.9%) and on the third day (0.8%). The symptom most frequently reported was dizziness (Table 3). All symptoms were reported to be mild and subsided within a period of 24 hours after onset.

**Table 2:** Age distribution of individuals reporting side-effects.

Age Group	Female	Male	Total
**<10**	19/455 (4.2%)	17/308 (5.5%)	36/763 (4.7%)
**10–19**	76/885 (8.6%)	50/663 (7.5%)	126/1548 (8.1%)
**20–29**	59/609 (9.7%)	32/281 (11.4%)	91/890 (10.2%)
**30–39**	85/477 (17.8%)	34/219 (15.5%)	119/696 (17.1%)
**40–49**	27/327 (8.3%)	28/211 (13.3%)	55/538 (10.2%)
**50+**	48/398 (12.0%)	29/222 (13.0%)	77/620 (12.4%)
**Total**	**314/3151 (10%)**	**190/1904 (10%)**	**504/5055 (10%)**

**Table 3:** Nature and timeline of side-effects during the pilot intervention.

Symptoms	Day 1	Day 2	Day 3	Day 4	Day 5	Day 6	Day 7	Total
Fever	15	6	3	0	0	0	0	24
Headache	59	8	1	0	0	0	0	68
Dizziness	160	13	1	0	0	0	0	174
Nausea	97	5	0	0	0	0	0	102
Vomiting	12	0	0	0	0	0	0	12
Diarrhoea	23	9	0	0	0	0	0	32
Abdominal Pain	106	21	0	0	0	0	0	127
Joint/Muscle Pain	19	2	0	0	0	0	0	21
Fatigue	24	7	0	0	0	0	0	31
Swelling of the limbs	0	2	0	0	0	0	0	2
Swelling of scrotum	0	0	0	0	0	0	0	0
Scrotal Pain	2	0	0	0	0	0	0	2
Rash	3	0	0	0	0	0	0	3
Itching	8	0	0	0	0	0	0	8
Others	9	0	0	0	0	0	0	9
**Total**	**537**	**73**	**5**	**0**	**0**	**0**	**0**	**615**

### The Country-Wide Intervention

As active surveillance for side-effects during the pilot intervention did not indicate any concern, two weeks after co-administration of the three drugs had taken place in Kinyasini and Mtambile, on December 2–3, 2006, the first large-scale triple drug co-administration was carried out in Zanzibar. This intervention was conducted under the newly established “Lymphatic Filariasis, Schistosomiasis and Soil-Transmitted Helminthiasis Integrated Programme” of the MoHSW. Every eligible individual in Zanzibar was targeted to receive the three drugs at the same time with the exception of those living in areas without transmission of schistosomiasis where only ivermectin and albendazole were administered. Such areas are restricted to Unguja island and include the whole Urban district of Stone Town, the whole South district, and some communities (shehias) in the North A district [18]–[20]. The same criteria for eligibility and ineligibility were applied as in the pilot intervention. Overall, about 700,000 individuals were administered three drugs (ivermectin, albendazole and praziquantel) and another 300,000 two drugs (ivermectin and albendazole).

As in the pilot intervention, the door-to-door strategy was chosen as the method of drug administration. For effective coverage, 4,161 drug distributors, used in previous LF-MDA interventions, were deployed and given extra training on how to use the two–sided drug pole in determining number of tablets. Each drug distributor was responsible for 50 households. All drug distributors were previously identified through a community-based participatory process, so as to guarantee their full acceptance by populations targeted. Many of them were health personnel or school teachers.

Zanzibar was divided into 14 MDA operational units – 9 of the units followed the administrative divisions (districts); 3 were the result of subdividing the urban districts into more manageable units; and the remaining 2 units targeted special groups within special institutions (SI) (soldiers, policemen, prisoners, etc.).

The social mobilization component was given high priority in the campaign. Three important areas were emphasized:

1. The use of drug distributors to carry out social mobilisation through two preparatory visits to the households. The distributor explained the rationale of the programme and prepared people for any potential side-effects; in addition, these visits were also intended to build rapport and confidence between the distributors and the household members.
2. The proactive involvement of the religious and political leaders of different parties, at national, regional, district and community level.
3. The effective use of mass media and other communication tools.

Similar to what had happened for the pilot intervention, both passive and active surveillance measures were taken during and after the country-wide drug administration. Passive surveillance was established during the intervention through the network of health centres and referral hospitals in both islands to monitor and respond to potential side-effects.

One week after the drug distribution, active surveillance measures were carried out to quantify the occurrence of side-effects in a sub-sample of the target population; the activity was incorporated into the routine survey intended to assess and check drug coverage. 35 communities (shehias) out of the 250 that form Zanzibar were randomly selected (20 in Unguja and 15 in Pemba), and 600–1000 people per site were interviewed, for a total of 19,043 individuals. A researcher was randomly assigned to each of the 35 evaluation sites and entered houses following a random route in their area. The same questionnaire as in the pilot intervention was used, and each individual visited was interviewed to check drug intake and investigate side-effects experienced.

## Results

Overall, only 266 individuals, equivalent to 1.4% of the interviewees who swallowed the drugs reported any side-effects, none of which was judged to be significant enough to justify a visit to the nearest health centre. All the side-effects were mild, the most frequent being fatigue (n = 102), abdominal pain (n = 67), dizziness (n = 57), fever (n = 27) and vomiting (n = 13). These side-effects were accepted and managed by those who reported them. They were transient and all counted for less than 24 hours. No difference in nature and frequency of side-effects was documented during the country-wide intervention between areas where ivermectin and albendazole only were distributed and areas where praziquantel was also added to the package.

## Discussion

The results of the pilot intervention in Kinyasini and Mtambile were considered representative of the worst possible epidemiological scenarios in Zanzibar and as such were deemed sufficient to justify the implementation of the first nationwide intervention, in which ivermectin and albendazole currently recommended for elimination of LF and praziquantel for control of schistosomiasis were administered at the same time. The first national scale triple therapy carried out in Africa or indeed globally.

Passive and active surveillance measures implemented during both the pilot and the country-wide intervention showed that side-effects experienced by individuals co-administered with the three drugs were mild and self-limiting events. It was not possible or feasible to obtain individual data on parasitological status hence this data does not allow us to establish a clear relationship between infection status and side-effects experienced. However, we believe that our data show that triple drug co-administration is a feasible option in real epidemiological scenarios such as those exemplified by Kinyasini and Mtambile, where pre-intervention prevalence rates for schistosomiasis and STH infections were high and where *W. bancrofti* micrfilaria prevalence remained above the 1% cut off point for MDA [17]. The studies using the ICT cards to measure antigenaemia have limited value at this stage of an LF programme as they only measure the presecnec of adult worm antigen. Their use and value in post MDA evaluation is in measuring the transmission to children born since the first MDA commenced.

The proportion of individuals reporting any side-effects in the pilot intervention phase (10%) is higher than that in the nationwide intervention phase (1.4%). This can be explained by the fact that Kinyasini and Mtambile are both sites with particularly high prevalence of helminthic infections, while the nationwide intervention also covered areas with lower prevalence. The two sites were specifically selected in order to assess the occurrence of side-effects in places where they are expected to be most frequent and most severe, so as to use the results of the pilot intervention as indicators and make a judgment before the implementation of the nationwide intervention. It is also possible, however, that the sensitivity of the surveillance system during the pilot intervention was higher than during the nationwide intervention: individuals responsible for surveillance during the pilot intervention - which had a research-like outlook - might have paid more attention to recording side-effects.

Overall, both in the pilot and the nationwide intervention, the number of individuals reporting side-effects following treatment registered a significant decline from that reported for distribution of ivermectin and albendazole only by 2002 (24%) [33], which could be explained by considering that the average wormload in infected individuals in 2002 may have been higher due to the fact that only two rounds of LF treatment had taken place, and in the two previous years (2000 and 2001) the second yearly round of albendazole for STH had not been implemented due to shortage of drugs.

Data from such a large population under study in Zanzibar therefore suggests that co-administration of the three drugs is a safe intervention when carried out in an area where LF, STH and schistosomiasis are co-endemic and where several rounds of treatment with one or two drugs have been implemented in the past.

However, it is necessary to emphasize the need for maintaining passive surveillance measures during similar interventions, and to ensure that detection, management and reporting of potential side-effects are a key component of any health intervention administering drugs [34].

There are opportunities arising from a coordinated approach to tackle multiple tropical diseases simultaneously. Currently many control/elimination programmes in Africa are constrained not by drug availability but by lack of the financial resources necessary for drug distribution, and it is expected that distribution costs will be lower when drugs are co-administered than in the case when several “vertical” interventions are conducted separately [35]–[38]. Meeting distribution costs would mean being able to implement control activities, since ivermectin and albendazole for LF elimination are donated. Praziquantel is not at presented donated on adequate scale to cover all the current needs.

In countries where there is a significant overlap between LF, STH and schistosomiasis [1], triple drug co-administration can be an option to cut down costs, boost control activities and improve the health status of neglected populations. Co-administration of anthelminthic drugs also offers an opportunity for integration of parasitic disease control programmes into the regular health system activities in Africa and elsewhere which has an appeal for most partners or donors. These interventions provide many benefits beyond purely disease elimination or control as they are relevant to the millennium development goals. MDA is a pro- poor non – discriminatory, and hence equitable intervention which reaches all eligible people irrespective of socio-economic status. This paper demonstrates co – administration of three highly efficacious antihelminthic drugs can be achieved at scale with very limited but acceptable side-effects. This work will pave the way for the next stage of studies in more intensely infected populations. This result will permit further expansion of the WHO policy of preventive chemotherapy [1] to needy populations for the control of neglected tropical diseases in sub Saharan Africa where extensive co-endemicity is the norm rather than the exception.
